# Role of Superconducting Materials in the Endeavor to Stop Climate Change and Reach Sustainable Development

**DOI:** 10.1007/s10948-023-06515-6

**Published:** 2023-02-03

**Authors:** M. Muralidhar, A. Sai Srikanth, S. Pinmangkorn, M. Santosh, J. Milos

**Affiliations:** 1 Materials for Energy and Environmental Laboratory, Superconducting Materials Group, Graduate School of Engineering and Science, SIT, 3-7-5 Toyosu, Koto City, Tokyo, 135-8548 Japan; 2 Institute of Physics, Na Slovance 2, Prague, CZ 18200 Czech Republic

**Keywords:** Global warming, Superconducting technology, GdYEr-123, MgB_2_, Critical current density

## Abstract

Progress in the mass production of newly developed bulk (Gd_0.33_Y_0.13_Er_0.53_)Ba_2_Cu_3_O_y_ “(Gd,Y,Er)123” and MgB_2_ systems is presented. Two batches of (Gd,Y,Er)123 pellets of 20 mm diameter and 7 mm thick were prepared in air by an infiltration growth “IG” process. Trapped field distribution profiles of fully grown bulk samples clearly showed that all samples were single-grain and the trapped field values were more than 0.5 T at 77 K, 1.3 mm above top surface. The best bulk exhibited the trapped field value of 0.63 T at 77 K. Ultra-sonication technique was employed for refining precursors of both (Gd,Y,Er)211 and boron. TEM studies revealed that boron powder subjected to ultrasonication was refined up to nanoscale. The micron-sized particles were reduced to nanoscale, which led to improvement of critical current by up to 36% in bulk MgB_2_ at 20 K and self-field. This progress in fabrication of high-performance LREBa_2_Cu_3_O_y_ and MgB_2_ superconducting bulks further promotes commercialization of superconductors’ production as a mode of sustainable technology.

## Introduction

**Fig. 1:**
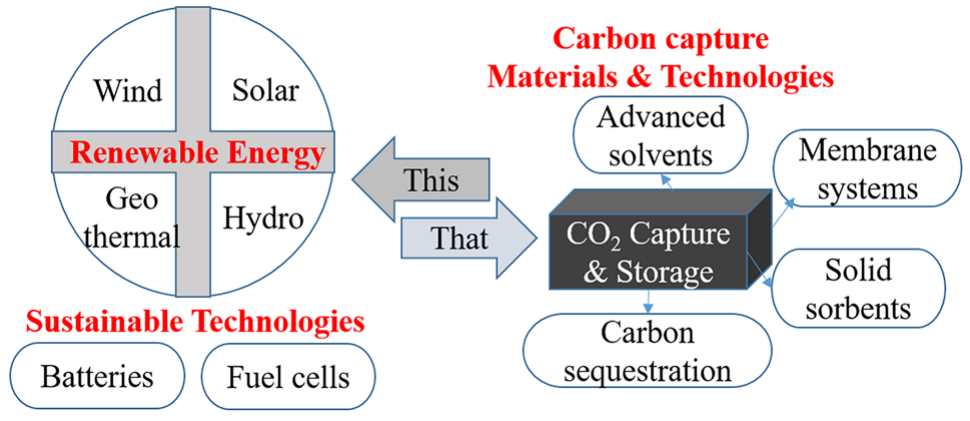
Current alternatives to address the impeding global warming and climate disruption

Climate change and global warming have an adverse impact on global life. Use of fossil fuels is responsible for excessive carbon dioxide emission into the atmosphere, leading to a steep rise in average global temperature. An extrapolation indicates the average global temperature rising by more than 1.5 °C until 2040, a limit set previously under the Paris Agreement, which would have horrendous implications [1]. World Meteorological Organization reported that concentration of CO_2_ reached 413.2 ppm in 2020 that accounted for 149% of pre-industrial level. Methane was 262% of the level of 1750, when human activities started to cause disrupting Earth’s natural equilibrium. A small decline in emissions due to COVID-19 did not have any discernible impact on atmospheric levels of greenhouse gasses and its growth rates [2]. In December 2021, we witnessed a potential breaking of ice shelf protecting the Thwaites Glacier in Antarctica as a consequence of warming ocean water. The melting and breakup of ice shelf can lead to opening of the Thwaites Glacier to warm ocean water. If breaking, it would open gateway to other glaciers it is holding. Just the melting of Thwaites Glacier can lead to a 2–3 feet rise of sea level, and if other glaciers included, the rise would be 10 feet [3]. Such a rise would not only change the coastline but also affect a huge amount of population especially in the coastal areas, flooding, sea life, vegetation, etc. All of the following incidents impel us to utilize green energy and sustainable technologies to reduce carbon footprint. Tremendous research enabled use of renewable energies like solar, wind, geo hydro, batteries, and several others [4]. In general, these attempts can be sorted in two groups, use of renewable energy and reduction of greenhouse gasses by capture technology as depicted in Fig. 1.

**Fig. 2:**
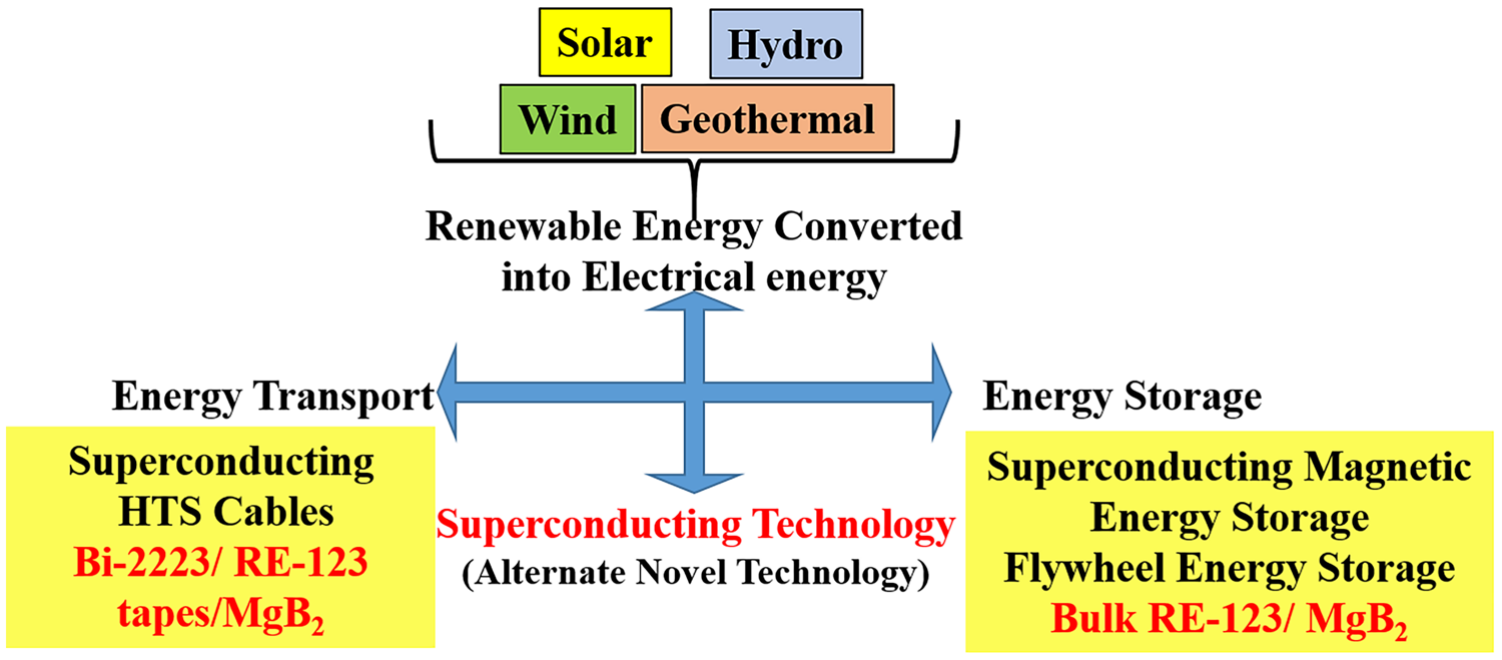
Superconducting technology for transportation as well as storage of renewable energy

Inger Andersen, Executive Director of UNEP stated in press release: “As the world looks to step up efforts to cut greenhouse gas emissions – efforts that are still not everywhere strong enough – it must also dramatically step up its game to adapt to climate changes,” which implies the need of holistic framework of intensive efforts and cutting-edge technologies to keep up with the timeline [5]. Lately, superconducting devices such as flywheel energy storage, fusion energy, and superconducting magnetic energy system (SMES) were intensively developed, despite their discovery long ago. The superconducting flywheel energy storage system stores electric energy as kinetic energy of a rotor suspended contactless on superconducting bearings. Kinetic energy of the flywheel can be converted to electric energy whenever needed. Fusion energy as a new clean energy source could be realized only after development of superconductors producing strong enough magnetic fields, up to 20 T. Hot plasma is enclosed and condensed by a huge magnetic field, which prevents its contact with any solid surface and is converted into electrical energy. Projects such as SPARC, ITER, and Tokamak are already in the final stage of realization [6–9]. SMES uses superconducting coils to carry loss less electric current and store its magnetic energy. It can serve in a large number (almost infinite) charge/discharge cycles with a high conversion efficiency of more than 95%. A SMES can roughly store 5 GWh, but requires a large amount of space for superconducting current loop (~ 0.5 miles) with cryogenic confinement [10]. Energy harvested in inhabited hot deserts or hot climate countries from renewable sources like sun or wind can be transferred without attenuation using superconducting cables over long distances to people in need. Evidently, superconducting technology can pave ways to harvesting clean energy at large scales using various systems. However, one of concerns is cost of installation and maintenance. Superconductors must be cheap, advanced in properties, and mass producible. In light of this theme, we developed a new ternary bulk high temperature rare-earth cuprate superconductor (REBa_2_Cu_3_O_7-x_ also known as REBCO or RE123) comprised of Gd, Dy, and Er via infiltration growth (IG) technique [11]. The main advantage of this ternary system with respect to already known ternary compounds (Nd,Eu,Gd)Ba_2_Cu_3_O_7-x_, (Nd,Sm,Gd)Ba_2_Cu_3_O_7-x_, (Sm,Eu,Gd)Ba_2_Cu_3_O_7-x_, etc. is its ability to be produced in ambient atmosphere [12–15]. We also incorporated a novel, high energy ultrasonic treatment to refine the precursor powder such as RE_2_BaCuO_5_ (RE211) at almost no cost which resulted in better flux pinning [15]. The connection of ultrasonically refined RE211 and IG method led to fine 211 secondary phase particles distributed uniformly in 123 matrix. Moreover, we have been able to batch-produce the bulks with consistent trapped magnetic field values, which is commercially attractive. The ultrasonic refinement technique was also effectively used in the fabrication process of bulk MgB_2_ superconductors. We were able to cost-efficiently increase the self-field *J*_*c*_ by 35% at 20 K, a common working temperature of MgB_2_ superconductors [16, 17]. Here, we present the summary of development and progress of bulk (Gd,Y,Er)Ba_2_Cu_3_O_7-x_ and bulk MgB_2_ superconductors toward making them cost-efficient by a technology addressing the climate issues and global warming (Fig. 2).

## Methods

### Production of (Gd_0.33_,Y_0.13_,Er_0.53_) Ba_2_Cu_3_O_y_ Samples

The Gd_2_BaCuO_5_ (Gd-211), Y_2_BaCuO_5_ (Y-211), Er_2_BaCuO_5_ (Er-211), ErBa_2_Cu_3_O_y_ (Er-123), and Ba_3_Cu_5_O_8_ precursors were prepared using high purity commercial powders of Gd_2_O_3_ (99.9%) and Er_2_O_3_ (99.9%) from NYC Co. Ltd., and Y_2_O_3_ (99.99%), BaCO_2_ (99%), and CuO (99.9%) from Kojundo Chem. Lab. Co. Ltd. The powders were mixed stoichiometrically to obtain the nominal composition of each powder. The mixed powders were heated up to maximum temperature at a heating rate of 100 °C/h, held there for 4 h, and then cooled to room temperature at cooling rate of -100 °C/h. Four calcinations at 820, 840, 860, and 880 °C were done to ensure a complete single-phase formation [13–15]. After every calcination, the powder was grinded for 2 h. In parallel, the powders of BaO_2_ and CuO were mixed in a nominal composition of Ba_3_Cu_5_O_8_ (Y-035) and calcined at 840 and 860 °C (12 h).

To reduce the particle size of calcined Gd211/Y211/Er211 powder, we used Mitsui ultrasonic homogenizer UX-300, while keeping constant power (300 W) and frequency (20 kHz). The 211 powder was dispersed in ethanol and ultra-sonicated for 80 min. We observed a substantial refinement of particles that will be discussed later. The ultra-sonicated Gd211, Y211, and Er211 powders were mixed in molar ratio of (Gd_0.33_Y_0.13_Er_0.53_)_2_BaCuO_5_ and 0.5 wt% of PtO_2_ was added to prepare the final homogeneous RE211 powder mixture. In parallel, a liquid phase (LP) was prepared of Er123 and Ba_3_Cu_5_O_8_ mixed in 1:1 ratio. The preforms were pelletized using a 20 mm diameter die under an applied pressure of 70 MPa. The secondary phase preform was placed on the LP preform. After that, Nd123 seed was placed at the top center of the secondary phase preform. The liquid phase preform was supported by Y_2_O_3_ and MgO single crystals to prevent liquid loss of LP during processing for achieving single-grain growth by infiltration growth (IG) process. The temperature profile for fabrication (Gd,Y,Er)123 bulk sample can be found elsewhere [11]. The oxygen annealing was done for 400 h at 500–375 °C in continuous oxygen flow (flow rate = 0.3 l/min).

### Production of MgB_2_

Cheap commercial boron (Furu-uchi chemicals, 300 mesh, 99% purity) was ultra-sonicated for 15 min and then mixed with amorphous Mg powder (99.9% purity, 200 meshes) in stoichiometric ratio. The powders were rigorously mixed and ground in a glove box to avoid oxidization. Then, pressing into 20 mm in diameter, 7 mm thick pellets followed. These pellets were immediately wrapped in titanium foils and heated at 775 °C for 3 h in a tube furnace with continuous argon flow [17, 18]. The pellets were then characterized and studied to make structure–property correlation.

### Characterization and Measurements

The microstructure of these samples was studied by field emission scanning electron microscope (FE-SEM, JEOL) as well as transmission electron microscope (TEM JEOL/JEM-2100). Superconducting critical temperature (*T*_*c*_) and magnetization hysteresis loops (*M*-*H*) were measured using SQUID Magnetometer (Quantum Design, model MPMS5). Specimens for SQUID measurements, with approximate dimensions of 1 × 1x0.75 mm^3^, were cut from bulk MgB_2_ and (Gd,Y,Er)123 samples. *J*_*c*_ was calculated from the *M*-*H* loops using the extended bean critical state model formula for finite rectangular samples [19],

1 $$J_c=20\triangle m/\lbrack a^2c\left(b-a/3\right)\rbrack$$

where *a* and *b* are cross-sectional dimensions, *b* > *a*, and c is thickness of the specimen (*a*, *b*, *c* in mm). Δ*m* (in emu units, 1 emu = 10^–3^ Am^2^) is the difference of magnetic moments during descending and ascending field in the *M*-*H* loop.

The trapped field (TF) for the (Gd,Y,Er)123 bulks was measured by field cooling method at 77.3 K under a field of 1 T. The Hall probe was placed at positions 0.3 (surface touched) and 1.3 mm above the top surface for scanning the TF value.

## Results and Discussion

In 2015, Railway Technical Research Institute (RTRI) completed one of the largest superconducting flywheel energy storage systems to that date, with energy storage capacity of 100 kWh, output of 300 kW, and maximum revolution speed of 6000 rpm [20]. To generate these numbers, high temperature superconducting bearings were used and the cryogenics was managed so that the maintenance expenses made this system outstanding. For harvesting more energy, especially in the form of solar and wind energies, huge areas must be used to setup the solar panels and/or windmills. Since these types of energy are geographic dependent, the best positions might be far away from the human colonies, like hot deserts and areas in inhabited regions [21]. In such cases to maximize the energy, we can use superconducting cables to transfer energy from remote areas to human colonies without attenuation losses. The same can be applicable to wind and hydro energies. Simultaneously, we can also employ superconductors to enhance the performance of application; for instance, a superconducting device employed in a wind mill motor can generate twice the power of a regular motor [22]. On the other hand, magnetic energy storage provided by superconductors with a fast response and long backup times is required for a successful transition from fossil fuels to wind and solar power. In case of transport, cables made of Bi-2212, Bi-2223, REBCO, and MgB_2_ superconductors were intensively studied [23]. Two main aspects of cables or tapes is the current carrying capacity and performance at high magnetic fields. Joints are also crucial, but will not be discussed here. We will present a survey of advancements in superconducting applications performed by various organizations as well as our own contribution.

### Cables

**Fig. 3:**
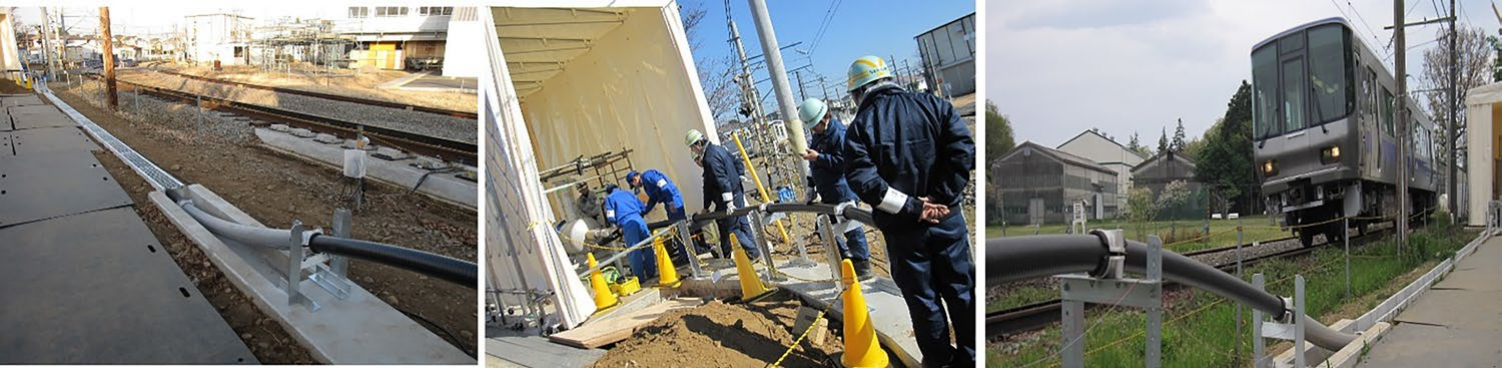
31 m Bi-2223 superconducting cable installation for train run test at RTRI, Tokyo

Superconducting cables have a great potential for many sectors such as power transfer, fault current limiters, Maglev, etc. The main challenge is to arrange transport of the energy harvested in deserted but rich of natural energy areas (like deserts, windy regions, and underground) to civilian societies. This looks to be a pipe dream, but with a considerable advancement in superconducting technology, it can become a reality. Due to high *T*_*c*_ they can be cooled to superconducting state using liquid nitrogen or by cryogen-free cryocoolers. They could be considered equivalent to optic fibers in high-speed information transfer. For example, the underground HTS power cable project issued to SuperPower Inc. at Albany, NY [24]. The installation was designed with 2 HTS cables using BSCCO as well as 2nd generation YBCO wire in length of 350 m. The 2nd generation wire showed much higher commercial value due to its inherent low cost and a better performance than 1st generation cables. Critical current of 1800 A (DC) was attained, and the cable was able to withstand high voltages. The president’s national energy policy mentions that superconductors are one of the promising technologies that will improve transmission, storage, and reliability of renewable energy [25]. Recent technological advances in the high-temperature superconducting underground power transmission cables will enable an increased access to all forms of energy, including renewable energy. These cables will allow 300% rise in capacity without excavations to lay new transmission lines. Another example is the use of superconducting technology to reduce the energy consumption in the railway systems. The superconducting cable allows to reduce energy consumption in electric railcars requiring a large amount of electric current to accelerate. RTRI began its dedicated research and development of superconducting cables for DC railway systems in 2008 [26, 27]. In 2009, Japan Science and Technology Agency (JST) and the Ministry of Land, Infrastructure in Japan started a national project, where a train running test was conducted with 31 m superconducting cable in the test track for the first time, followed by a 310 m superconducting cable and its initialization in test track presented in Fig. 3 [28, 29]. Efforts are underway for the installation of 1 km track in Tokyo area. Simulations estimated that use of superconducting cables could save 5% energy per day on an average general city railway model. This is because of cryogenic cooling energy is less than the amount of regeneration and joule heat loss. One of the main challenges in this technology is the high expense of cryogenic technology and long-distance cryogenics pumping. It has to be solved with the advancements in that field. In any case, the superconducting cables are crucial to stop and reverse the climate change.

MgB_2_ cables are commercially attractive with only 20% of the cost of HTS coated conductors, but one has to use liquid helium or hydrogen as cryogenic fluid, which is expensive and limits the material’s use [23]. With the increase in demand and consumption of energy resources by power-hungry distant human societies, environmentally friendly superconducting HTS DC transmissions lines are crucial for linking abundant renewable and green energies. Advances in performance of these materials, cost of their fabrication, and the cryogenics are crucial to make the superconducting cable a cost-effective alternative to current overhead transmission lines which are far from being environmentally friendly.

### (Gd,Y,Er)Ba_2_Cu_3_O_y_ Bulk System

**Fig. 4:**
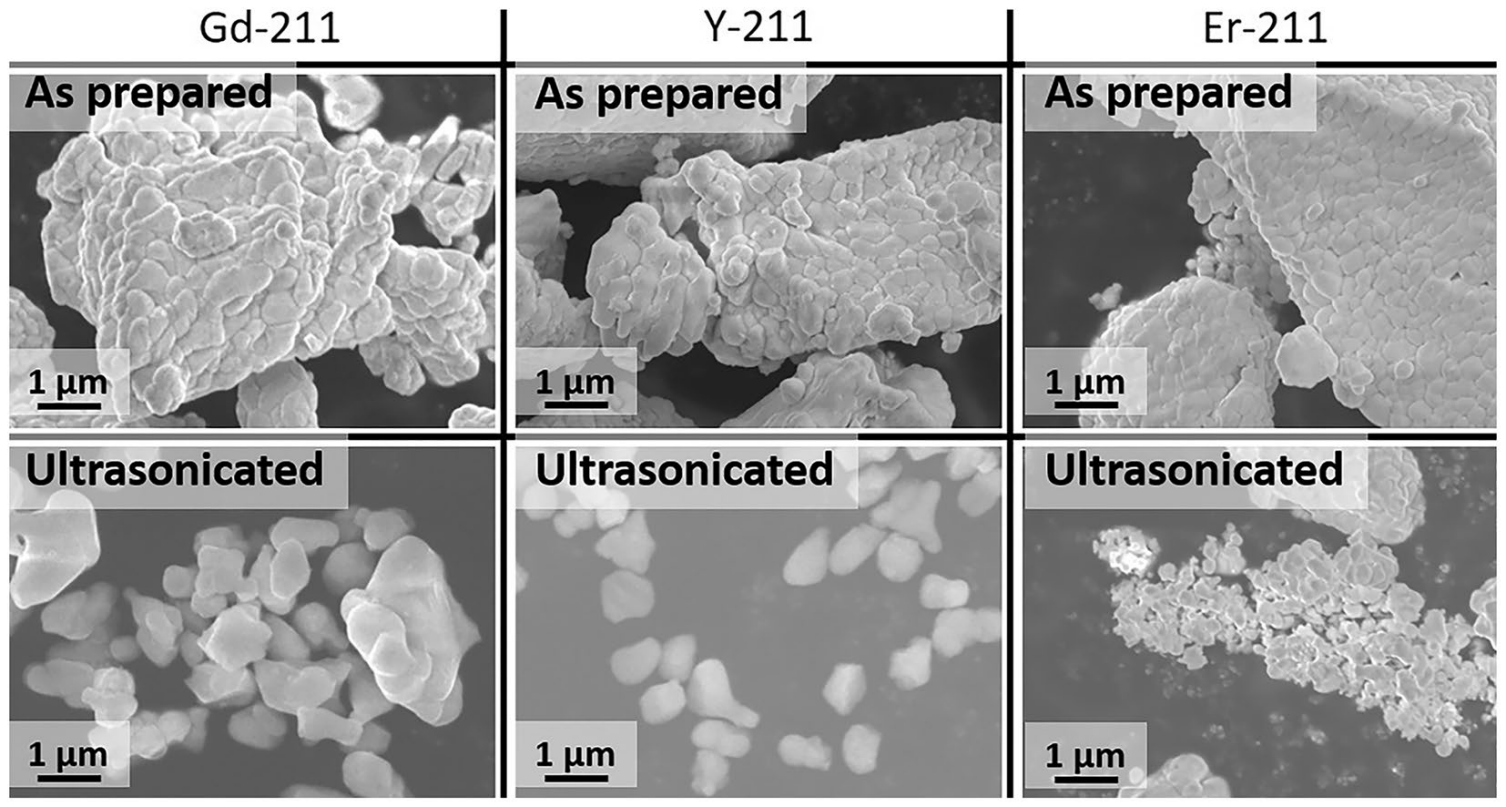
FE-SEM images of as prepared RE-211 powders (RE – Gd, Y, Er) via calcination before and after ultrasonication

We first ultrasonicated Gd211, Y211, and Er211 powders to refine them into nanoscale. Figure 4 shows FE-SEM images of prepared and ultra-sonicated (for 80 min) Gd211, Y211, and Er211 powders. One can observe that the as-prepared 211 powders consisted of particles larger than 5 µm, sometimes agglomerated, while the ultra-sonicated 211 powders had much smaller particles, better dispersed. The average particle size of refined Gd211, Y211, and Er211 powders was 500, 600, and 200 nm, respectively. The fabrication technique employed was infiltration growth (IG) as known to ensure better distribution of 211 in the bulk, resulting in more uniform properties. Although it was not perfectly uniform, the distribution was much better than with conventional melt growth (MG) technique. In addition, IG process tends to produce high density bulks with a reduced amount of pores and no shrinkage [30, 31]. Initially, we optimized the composition and arrived at an optimum combination of RE elements for maximum TF and *J*_*c*_.

**Fig. 5:**
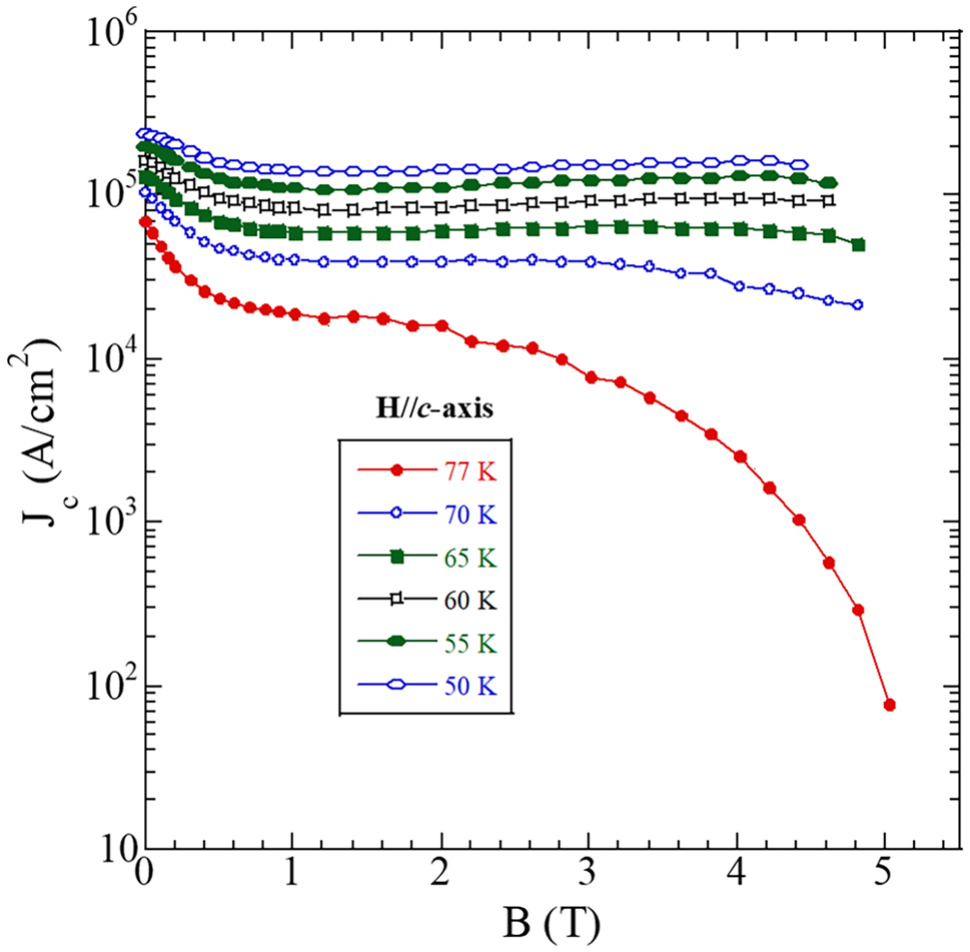
Semi-logarithmic dependence of *J*_*c*_ on applied magnet field of Er-rich GdYEr-123 bulk at various working temperatures

**Fig. 6:**
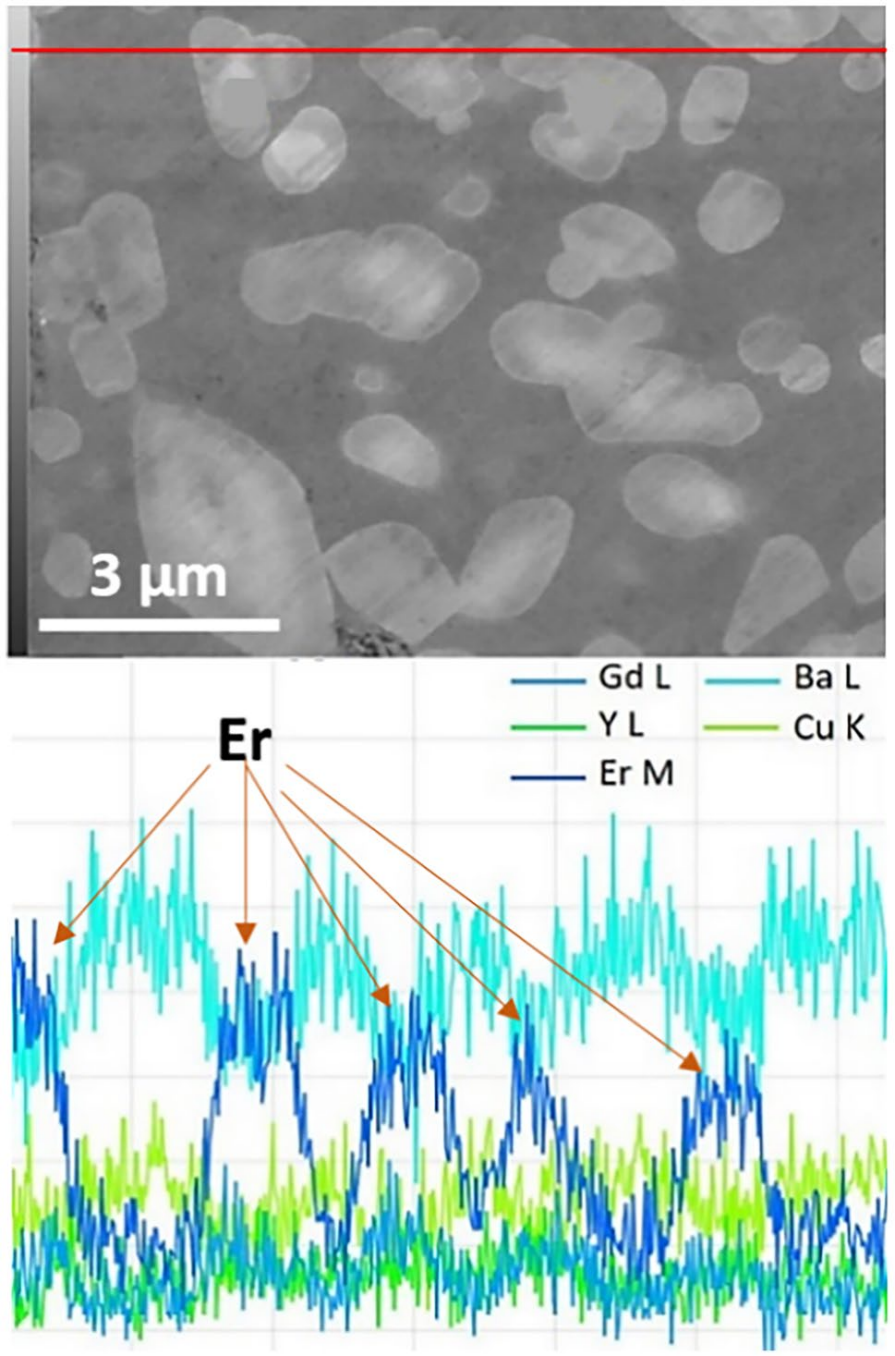
EDX line scan on (Gd,Y,Er)BCO ternary bulk. White and dark areas correspond to (Gd,Y,Er)211 particles and the (Gd,Y,Er)123 matrix, respectively

Our earlier studies showed that Er-rich system (Gd_0.33_Y_0.33-x_Er_0.33 + x_)-123 bulk superconductor with *x* = 0.2 had the best properties [11]. Figure 5 shows the semilogarithmic plot of the *J*_*c*_(*B*) dependence at various temperatures. The *J*_*c*_ at 4–5 T was close to 10^5^ A/cm^2^ at temperature below 65 K. These results point out a wide range of applicability of the current-optimized (Gd,Y,Er)123 at various temperatures and fields. The *J*_*c*_ value of the bulk (Gd,Y,Er)123 superconductor prepared by IG process is higher than in binary (Gd,Y)123 [32], as well as single-element Y-123 system [33, 34] at self-field, high field, and various temperatures. This superior performance is based on the microstructure, where many tiny 211 particles, usually Er-rich, are responsible for enhancing flux pinning as indicated by EDX. We performed an EDX line scan of the microstructure so that it includes both (Gd,Y,Er)211 secondary phase particles as well as (Gd,Y,Er)123 matrix (see Fig. 6). Different colors of spectra correspond to different elements. According to the spectral analysis, the dark areas (supposedly matrix) comprise of Gd, Y, and Er in equal ratio, whereas the bright areas (RE211) are Er-rich. Quantitative analysis of the microstructure can be found elsewhere [11].

**Fig. 7:**
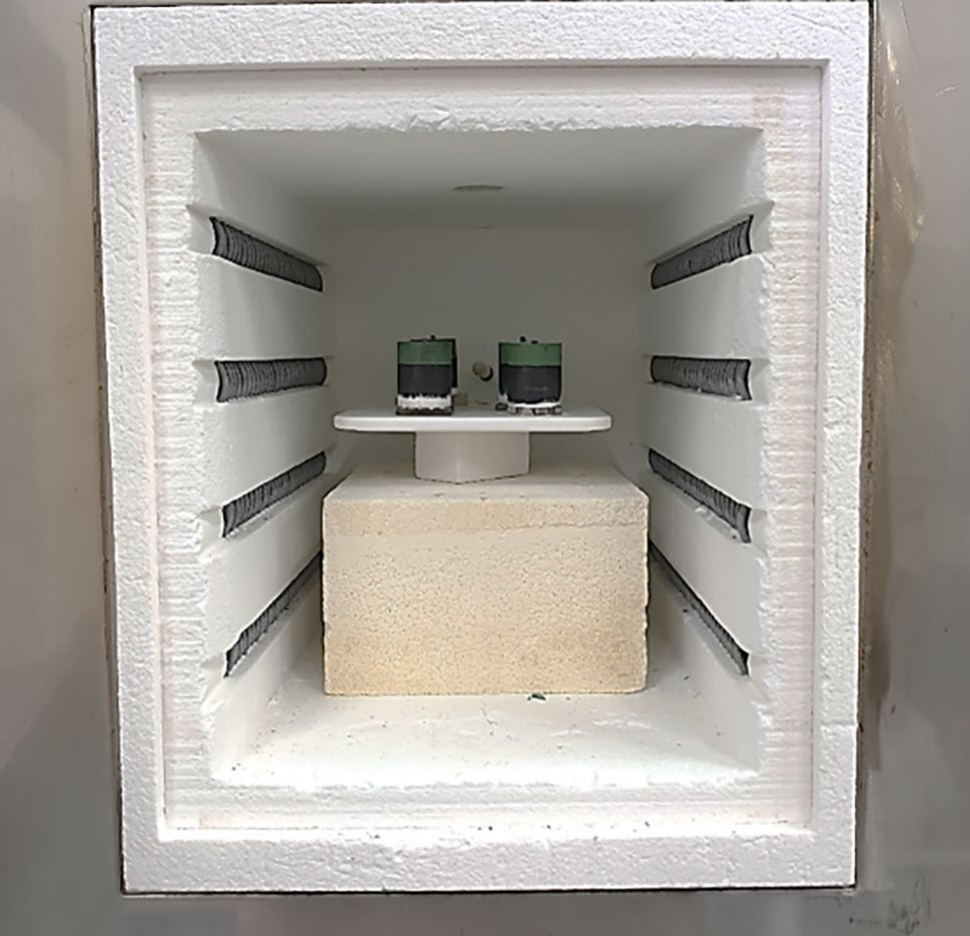
Demonstration of batch production of (Gd,Y,Er)BCO ternary bulks via IG technique in a small box furnace (21 × 15 × 16 cm.^3^)

**Fig. 8:**
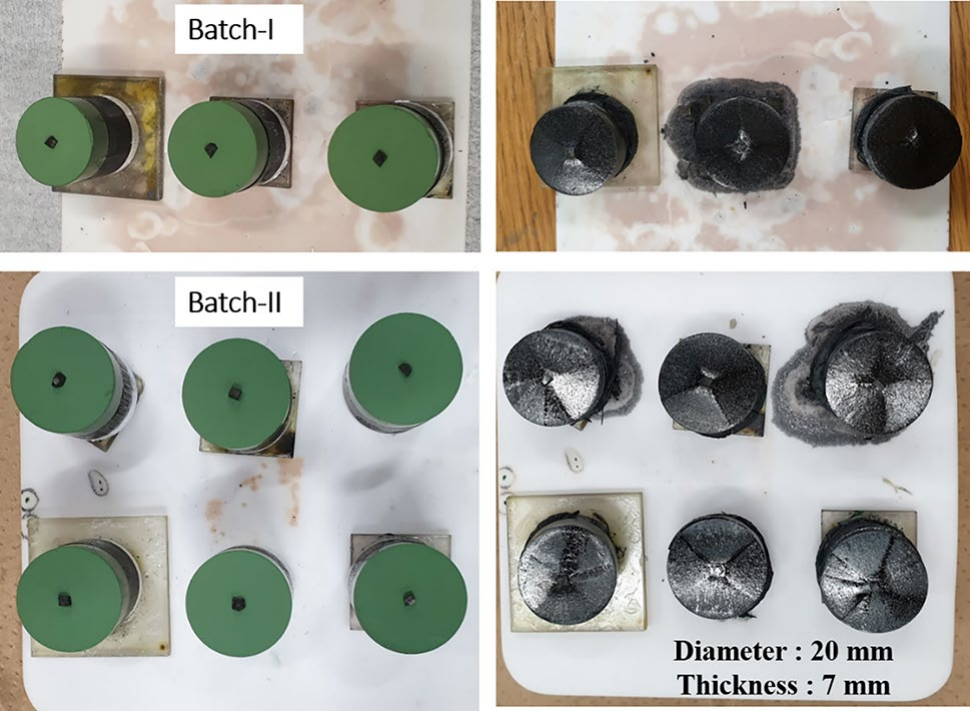
Batches of IG preforms of (GdYEr)211 stacked over liquid phase on the left; fully grown 20 mm in diameter and 7 mm thick single grained bulk (GdYEr)123 superconducting on the right

**Fig. 9:**
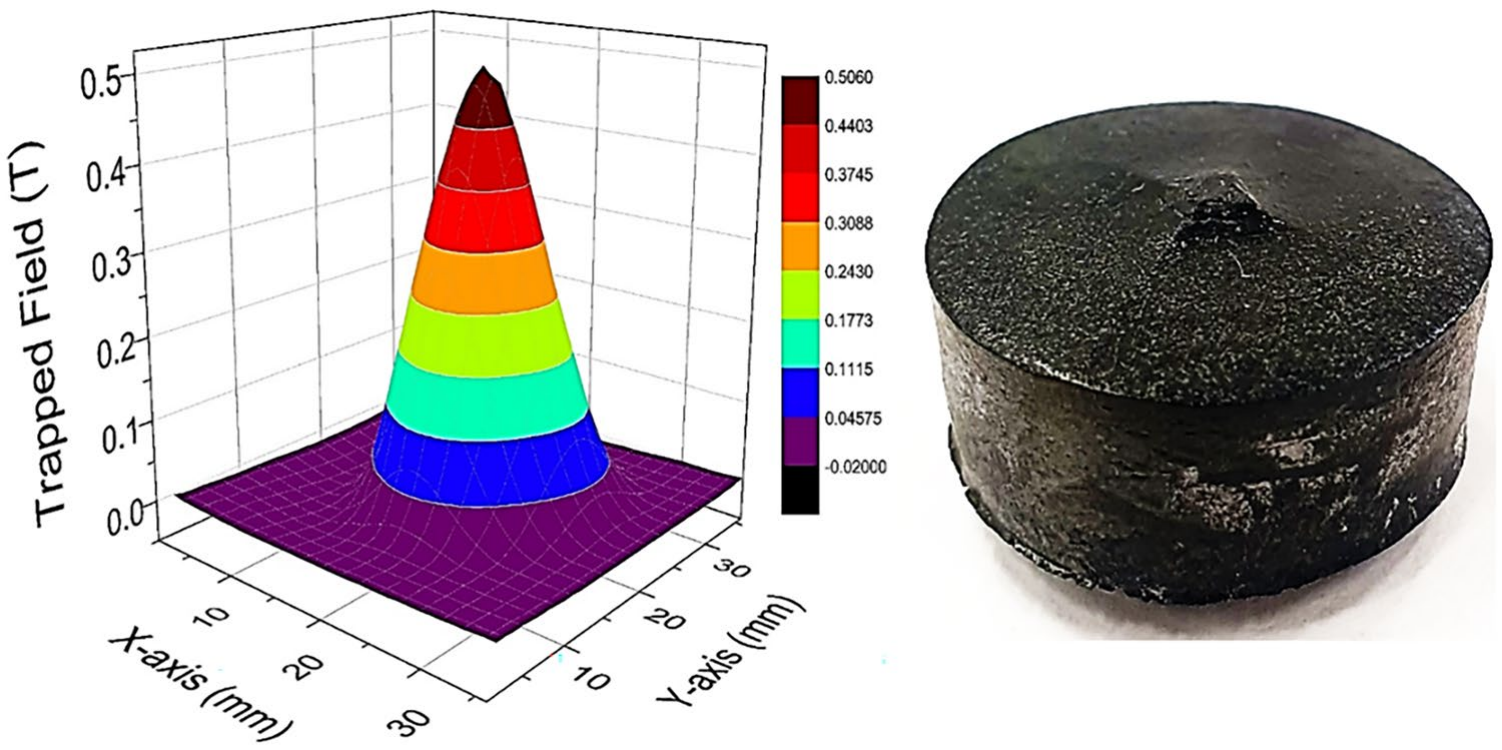
Trapped field behavior of bulk (GdYEr)123 superconductors measure at 1.3 mm away from the sample surface

**Fig. 10:**
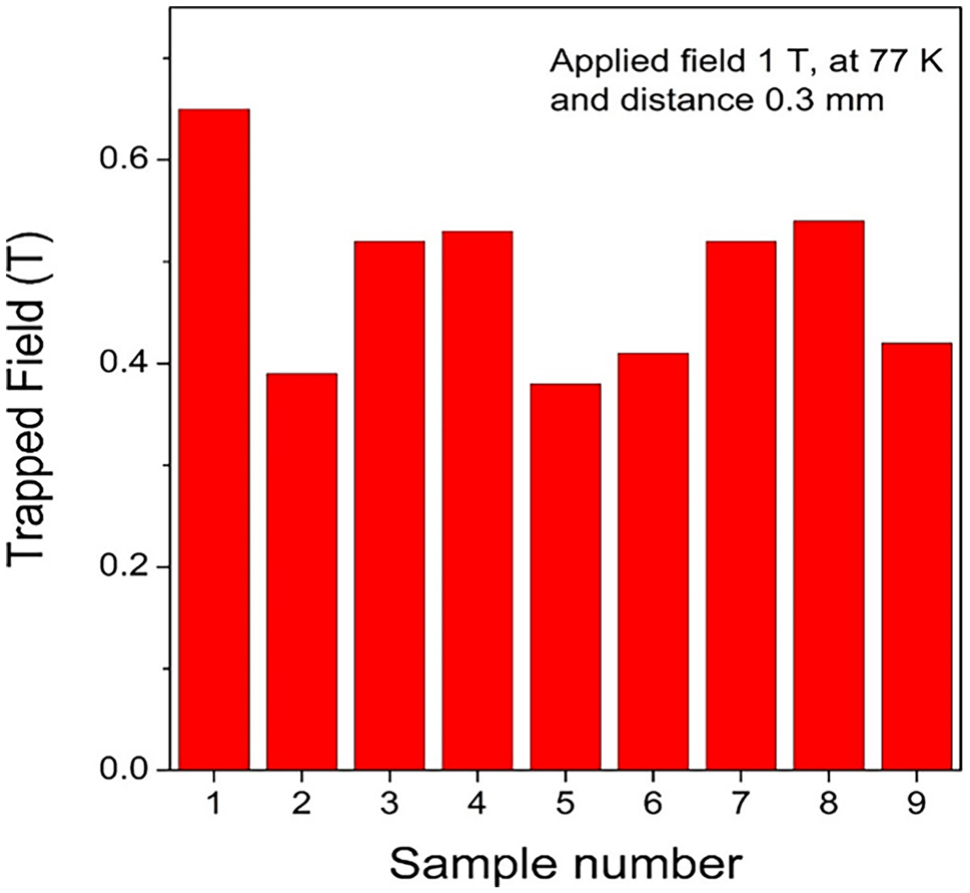
Trapped field values of batch produced bulk (GdyEr)123 at 0.3 mm away from the surface at 77 K

Superior superconducting properties at 77.3 K and 90.2 K of ternary systems (Nd,Eu,Gd)123, (Nd,Sm,Gd)123, (Sm,Eu,Gd)123, etc. were reported in Ref. [13, 14, 35]. In this case, the samples were fabricated in a special, oxygen controlled, environment, which complicated the process and increased the cost for mass production. The current specimens of (Gd,Y,Er)-123 system are processes in ambient atmosphere, which makes them easy to be mass produced. We repeatedly batch processed (Gd,Y,Er)-123 pellets at laboratory scale in a small commercial box furnace (21 × 15 × 16 cm^3^) (Fig. 7). Three and six bulks were fabricated in the batches labeled I and II. Figure 8 shows the completely grown (Gd,Y,Er)123 bulks with perfect fourfold growth structure. The 20 mm in diameter and 7 mm thick samples were tested at 77.3 K for trapped field using a 1 T electromagnet. The best sample showed a high-trapped field value of 0.65 T at 0.3 mm distance from the probe (surface touched). The TF was also measured 1.3 mm away from the probe, exhibiting a beautiful cone shape as shown in Fig. 9. In all other bulks, TF > 0.4 T and in more than 50% of the bulks TF > 0.5 T (Fig. 10). Although there is a slight variation in TF, it is mostly due to the variation in bulk thickness and heat distribution in the furnace. Using a large specially designed furnace can solve such issues. REBCO bulks have been proven to be an upgrade for the flywheel energy storage as a green sustainable technology. With YBCO superconducting magnets, the flywheel rotation speed proved to reach 7,500 rpm, enhancing energy storage to ~ 2.24 kWh, with very minute temperature rise of the stator (< 20 mK) after ~ 6 h operation [36]. These bulks used in superconducting magnetic bearings (SMB) are suitable for a 10 kWh-class flywheel system. The YBCO ring bulks are stacked up to make the SMB. The individual TF was around 0.4–0.7 T [37]. The current ternary GdYEr-123 bulks with size 20 mm by 7 mm having the TF values close to high end of the required TF are ideal candidates for the Superconducting Flywheel Energy Storage scenario. Production of blocks around 40–50 mm in diameter is next step toward real applications.

### MgB_2_ System

**Fig. 11:**
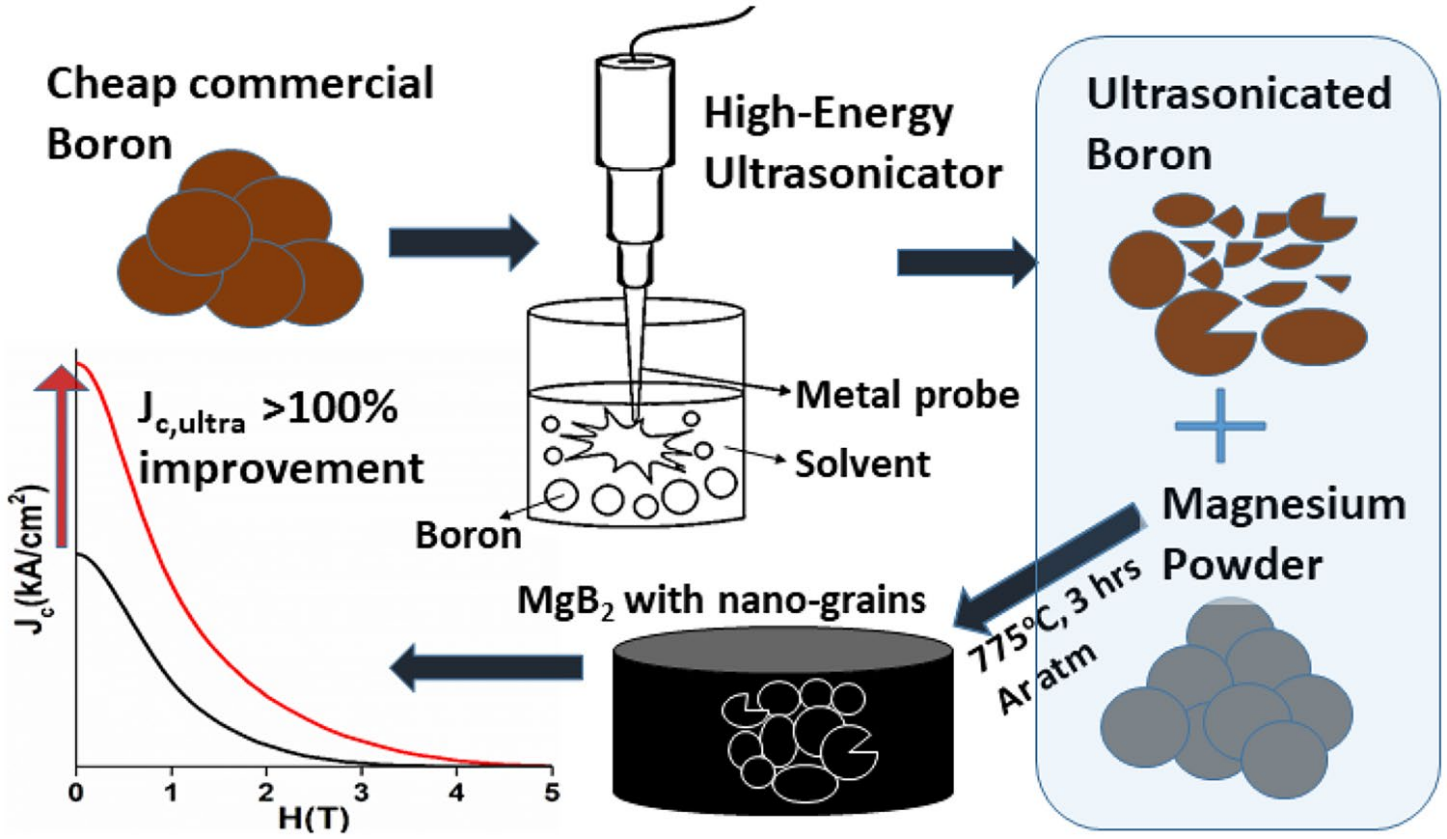
Depiction of ultrasonic refinement of cheap large boron to nano-boron

**Fig. 12:**
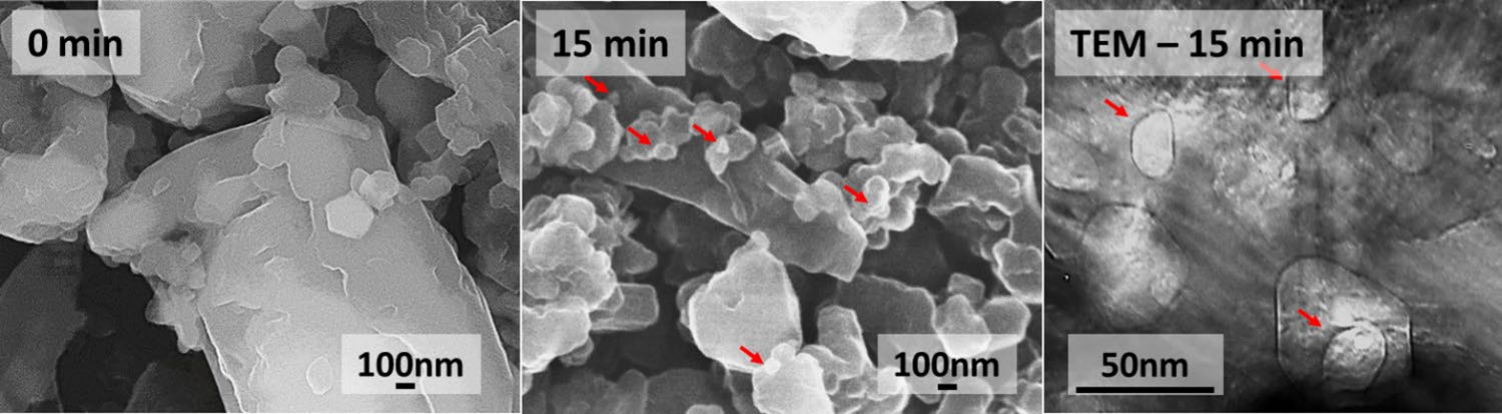
FE-SEM images of cheap boron before (in the left) and after (in the center) ultra-sonication. TEM micrographs indicating nano-sized boron powder (in the right)

MgB_2_ bulk system is very interesting due to its simple and fast fabrication compared to REBCO. Despite its moderate *T*_c_ at 39 K, it is preferred for certain applications [38]. Intricate shapes can be sintered from MgB_2_ superconductors, which is difficult with other systems, which require textured morphology for showing good properties. For instance, MgB_2_ bulk cylinders up to 65 mm in diameter and 100 mm height produced by reactive Mg-liquid infiltration technique are ideal superconductors for passive magnetic bearings in flywheel energy storage or other rotating electrical energy systems [39]. Recently, 40 mm in size, high-density MgB_2_ bulk was produced by spark plasma sintering technique [40]. After years of phase diagram analyses and trials, the optimum synthesis parameters for high *J*_*c*_ were identified. We also understood the importance of microstructural control in enhancing the *J*_*c*_ of bulk MgB_2_ [41]. We carried out experiments to optimize sintering conditions, tested the sintering temperature range from 700 to 900 °C, and achieved *J*_*c*_ of 1.8 × 10^5^ A/cm^2^ at 20 K, 0 T when the sintering temperatures were around 775 – 800 °C [18]. We note that this *J*_*c*_ value is comparable or better than that of ternary HTSC at 77 K. To enhance grain boundary pinning in bulk MgB_2_, we used a commercial nano-amorphous boron in one of our previous works. The *J*_*c*_ results were outstanding, with the value of 4.1 × 10^5^ A/cm^2^. However, the commercial nano-amorphous boron powder was expensive, making the fabrication process costly. A similar situation was also with carbon-encapsulated boron precursor (PAVEZYUM, Belgium) [42]. It was almost three times more expensive than the conventional boron powder (also called here as cheap commercial boron powder, “CCBP”). In the light of cheap mass production of bulk MgB_2_ for commercial applications, we tested some novel methods. High-energy ultrasonication was employed to refine conventional cheap boron powder. In this technique, powerful waves are generated using vibration from a metal probe, which causes a turbulence in the medium and passes high energy to the particles that bombard each other and container walls, resulting in a particle splintering (see Fig. 11). Tiny air bubbles are formed, releasing destructive forces inside the solution, which aid in the particles splintering. An important advantage of this method is that waves’ generation can be controlled and optimized by tuning frequency and power. We were able to successfully reduce the micron sized boron particles to nano-powders using Mitsui UX-300 Ultrasonic Homogenizer (constant power at 50% (150 W) and frequency at 20 kHz) as confirmed by FE-SEM as well as TEM studies (Fig. 12). In this way, we were able to self-produce a cheap nano-boron powder capable to replace the commercial nano-boron.

**Fig. 13:**
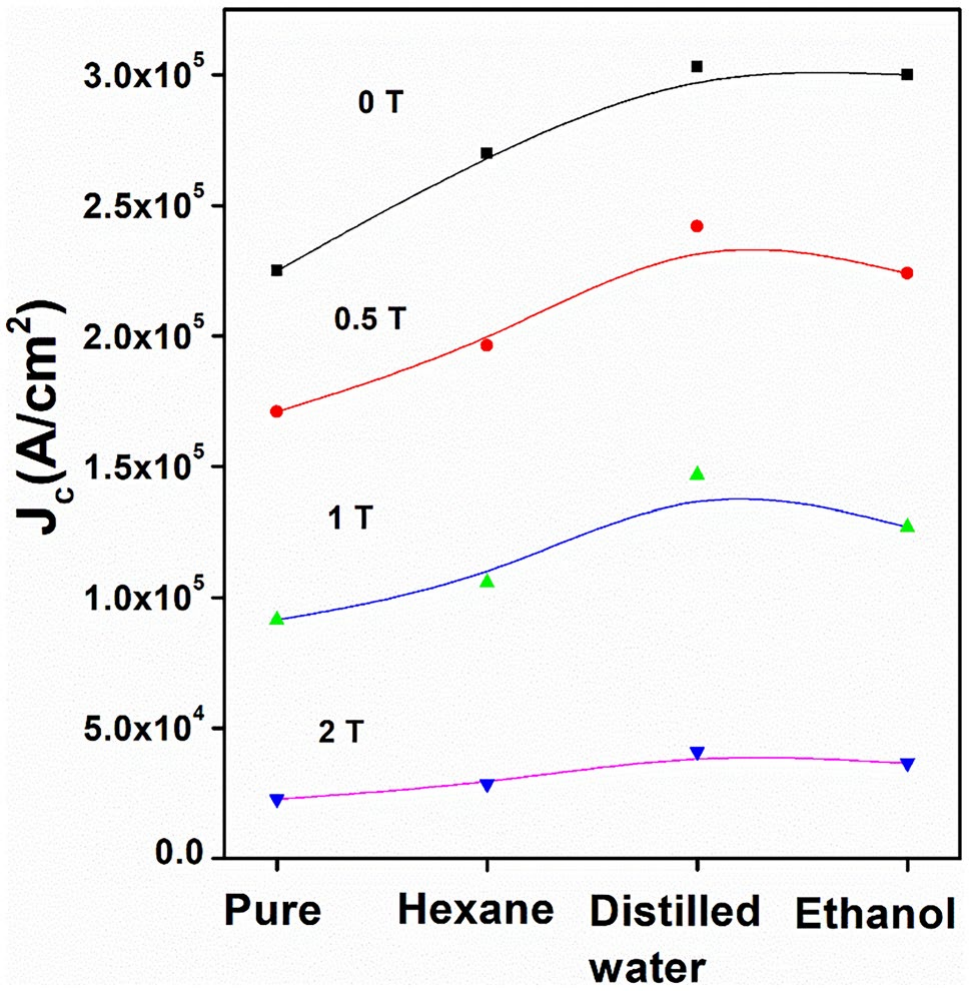
Critical current densities (*J*_*c*_) of MgB_2_ prepared from boron ultra-sonicated in different media at various fields

This ultra-sonicated nano-boron powder was used to fabricate bulk MgB_2_. So far, we have achieved *J*_*c*_ of 3 × 10^5^ A/cm^2^. Interestingly, we found that ultra-sonication medium is also an important factor. Hence, we have tested the ultra-sonication in media such as ethanol, hexane, and distilled water. In these experiments, we observed 36, 20, and 36% enhancements in the self-field *J*_*c*_, respectively. Figure 13 shows the *J*_*c*_ of MgB_2_ bulks at various fields. The nano-sized grains in the bulk matrix led to increased grain boundary pinning that is most effective at low magnetic fields. More information on supporting microstructural evidences can be found in Refs. [16, 17]. The development of superconducting materials and its performance are crucial for stopping the climate change and contributing to UN’s Sustainable Development Goal by 2030. Superconducting bulk materials and cables will address the issue of global warming by enabling green energy transfer. This will contribute to the crucial task to stop rise of global average temperature to secure our future on this planet.

## Conclusions

The role of superconductors in a sustainable technology is crucial due to the impending climate disruption caused by greenhouse emissions. Superconductors play a promising role in loss-less energy transportation as well as storage, which are important to efficiently utilize the power from renewable energy sources. HTS materials’ performance and low-cost fabrication steadily advance. We show the progress in new ternary GdYEr-123 system, which is mass producible in air. We successfully fabricated 9 fully-grown bulk specimens in two batches using IG technique. Most of these bulks showed TF > 0.5 T at 77 K when magnetized using a 1 T electro-magnet. The best bulk exhibited 0.63 T at 77 K with a beautiful cone-shaped profile. The work shows the capacity to produce such high TF ternary GdYEr-123 bulks at industrial scale, while the system can be further optimized by exploring various RE components. In addition, the 211 powder can be subjected to ultra-sonication that refines it leading to fine Er-rich 211 particles in the final matrix. It enhances flux pinning and improves the performance. The ultra-sonication technique was also employed in the refining boron and enhancing the properties of bulk MgB_2_ fabricated fast at low cost. We confirmed with TEM reduction of the micron sized particles to nano-scale. These production methods are cost efficient techniques that can lead to enhancement in performance, paving path to commercialization of the superconductors as a sustainable technology.
